# Topical Pioglitazone Nanoformulation for the Treatment of Atopic Dermatitis: Design, Characterization and Efficacy in Hairless Mouse Model

**DOI:** 10.3390/pharmaceutics12030255

**Published:** 2020-03-12

**Authors:** Lupe Carolina Espinoza, Rodrigo Vera-García, Marcelle Silva-Abreu, Òscar Domènech, Josefa Badia, María J. Rodríguez-Lagunas, Beatriz Clares, Ana Cristina Calpena

**Affiliations:** 1 Department of Pharmacy, Pharmaceutical Technology and Physical Chemistry, Faculty of Pharmacy and Food Sciences, University of Barcelona, 08028 Barcelona, Spain; marcellesabreu@gmail.com (M.S.-A.); odomenech@ub.edu (Ò.D.);; 2 Departamento de Química y Ciencias Exactas, Universidad Técnica Particular de Loja, Loja 1101608, Ecuador; 3 Department of Biochemistry and Physiology, Faculty of Pharmacy and Food Sciences, University of Barcelona, 08028 Barcelona, Spain; rodrigo.vera.garcia@ub.edu (R.V.-G.); josefabadia@ub.edu (J.B.); mjrodriguez@ub.edu (M.J.R.-L.); 4 Institute of Biomedicine, University of Barcelona, 08028 Barcelona, Spain; 5 Institute of Nanoscience and Nanotechnology (IN2UB), University of Barcelona, 08028 Barcelona, Spain; beatrizclares@ugr.es; 6 Nutrition and Food Safety Research Institute (INSA-UB), 08921 Santa Coloma de Gramenet, Spain; 7 Department of Pharmacy and Pharmaceutical Technology, Faculty of Pharmacy, University of Granada, 18002 Granada, Spain

**Keywords:** atopic dermatitis, pioglitazone, nanoemulsion, Pluronic F127

## Abstract

Pioglitazone (PGZ) is a drug used to treat type 2 diabetes mellitus that has been reported to show additional therapeutic activities on diverse inflammatory parameters. The aim of this study was to optimize a topical PGZ-loaded nanoemulsion (PGZ-NE) in order to evaluate its effectiveness for treating atopic dermatitis (AD). The composition of the nanoformulation was established by pseudo-ternary diagram. Parameters such as physical properties, stability, in vitro release profile, and ex vivo permeation were determined. The efficacy study was carried out using oxazolone-induced AD model in hairless mice. PGZ-NE released the drug following a hyperbolic kinetic. Additionally, its properties provided high retention potential of drug inside the skin. Therapeutic benefits of PGZ-NE were confirmed on diverse events of the inflammatory process, such as reduction of lesions, enhancement of skin barrier function, diminished infiltration of inflammatory cells, and expression of pro-inflammatory cytokines. These results were reinforced by atomic force microscope (AFM), which demonstrated the ability of the formulation to revert the rigidification caused by oxazolone and consequently improve the elasticity of the skin. These results suggest that PGZ-NE may be a promising treatment for inflammatory dermatological conditions such as AD.

## 1. Introduction

Atopic dermatitis (AD) is commonly referred to as atopic eczema and is a chronically relapsing, inflammatory, and intensely pruritic skin disease that affects up to 20% of children and 1–3% of adults [1]. It is caused by a complex interaction among genetic predispositions, environmental factors, and dysregulation of the immune system. Symptoms of AD include: a fluctuating course, hyperactive immune response to environmental factors, skin barrier dysfunction, drying, itching, and highly inflamed skin lesions [2,3]. These symptoms can provoke psychological distress and functional disturbances that significantly impact the quality of life of patients [4]. The molecular mechanisms underlying the visible signs of the disease are highly diverse, including the immunological activation of a variety of T helper (Th) cells such as Th1, Th2, Th17, and Th22 accompanied by defective epidermal barrier functionality. The clinical features depend on the age and the phase of the disease (acute or chronic). An overexpression of inflammation markers associated with Th17 have been observed in pediatric, Asian, intrinsic, and elderly patients with AD [5,6]. Th17 activation stimulates the production of Th2-associated cytokines and induces skin barrier impairment, therefore Th17 targeting could be used as an effective strategy to treat AD among these patient groups [3].

Diagnostic criteria of AD are based on clinical observation, allergy tests, and systematically ruling out other skin disorders [5]. AD treatment is focused on reducing itching, restoring skin, and attenuating the inflammation. To that end, patients with AD require topical treatment with emollients to provide soothing and moisturizing effects in addition to anti-inflammatory therapies with topical calcineurin inhibitors or glucocorticosteroids. In severe cases, systemic treatment using glucocorticoids, cyclosporine A, azathioprine, and mycophenolate mofetil may be required [7]. However, systemic therapy shows serious adverse effects and has limited efficacy in many patients in whom the symptoms often reappear after discontinuing treatment [8]. Conventional topical treatment (creams, ointments, and lotions) shows limited accessibility into the skin. Thus, there are unsatisfied needs for novel topical formulations that penetrate deep into the epidermis to provide more effective treatment of AD [9,10].

The incorporation of drugs into a nanoemulsion (NE) could be used as a strategy to facilitate its topical application and improve the permeation of drug into the skin due to its content of oil and surfactants, nanometric droplet size, and high solubilizing potential for lipophilic and hydrophilic drugs [11,12]. Moreover, an additional advantage could be provided by the addition of a polymer in the aqueous phase of NE in order to overcome its low viscosity and consequently improve its appearance, facilitate its application, increase its residence time at the site of action, and improve dermal bioavailability of the drug [13,14]. Amphiphilic block co-polymers are used as pharmaceutical excipients in several dosage forms because the chemical flexibility of their structure allows the development of versatile drug carriers [15,16].

Pioglitazone-HCl (PGZ) is a member of the thiazolidinediones and is used to treat type 2 diabetes mellitus (T2DM) due to its efficacy in glycemic control by acting as an insulin sensitizer. PGZ is categorized as a class II drug according to the Biopharmaceutical Classification System, and has a molecular weight of 392.9 g/mol, pK_a_ of 5.8 and 6.8, and log*P* of 2.3 [17]. PGZ is a selective agonist of peroxisome proliferator-activated receptor γ (PPAR γ), which is a nuclear transcription factor that modulates the expression of multiple genes, regulates metabolic processes (lipid and glucose metabolism), and reduces inflammatory responses [18]. PGZ therapy in animal models, patients with T2DM, and obese subjects has shown beneficial effects on diverse inflammatory parameters such as reduced infiltration of macrophages, neutrophils, and dendritic cells as well as suppressed expression of Tumor necrosis factor-alpha (TNF-α), Interleukin-6 (IL-6), and Interleukin-17 (IL-17) [19,20]. Based on these findings, it is evident that the therapeutic activities of PGZ go far beyond its role on glycemic control and suggest that it could be a promising candidate for the treatment of inflammatory dermatological conditions [21].

The aim of this study was to develop a PGZ-loaded nanoemulsion (PGZ-NE) and investigate its effect on clinical, histological, and immunological aspects of AD using oxazolone-sensitized hairless mouse model.

## 2. Materials and Methods

### 2.1. Materials

PGZ was obtained from Capot Chemical (Hangzhou, China). Capryol 90 (propylene glycol monocaprylate), Labrasol (caprylocaproyl polyoxyl-8 glycerides), and Transcutol-P (diethylene glycol monoethyl ether) were provided by Gattefossé (Saint-Priest, France). Pluronic F127 (Poloxamer 407) was obtained from Fragon (Barcelona, Spain). Oxazolone was purchased from Sigma-Aldrich (Darmstadt, Germany). Components for histological analysis were purchased from Sigma and Thermo Fisher Scientific (Barcelona, Spain). Ultrapure water was obtained using a Millipore Milli-Q purification system (Millipore Corporation; Burlington, MA). All reagents were of analytical grade.

### 2.2. Pseudo-Ternary Phase Diagrams and Preparation of PGZ-NE

The components used in the construction of the pseudo-ternary phase diagram were selected based on their solubilizing capacity for PGZ. Reported results of this parameter were used and an additional determination for Capryol 90 was performed following the prescribed method [21]. Capryol 90 was selected as the oil phase, Labrasol as surfactant, Transcutol-P as cosurfactant, and water as the aqueous phase. Labrasol and Transcutol-P (Smix) were used at the ratio 1:2. To determine the boundaries of diagram, oil and Smix were mixed at the ratios from 9:1 to 1:9 (*w/w*) while water was added to each mixture by titration method until turbidity or phase separation. PGZ-NE (1 mg/mL) was prepared by incorporating PGZ in oil phase under stirring at 700 rpm for 15 min, after which Labrasol and Transcutol-P were incorporated by continuous stirring for 20 min. Finally, the solution of Pluronic F127 dissolved in water was added under the same condition of stirring for 15 min.

### 2.3. Characterization of PGZ-NE

Calibrated digital pH meter GLP 22 (Crison Instruments; Barcelona, Spain) was used to determine the pH of PGZ-NE.

The droplet size and polydispersity index of the formulation were evaluated without dilution after 24 h of preparation at 25 °C by dynamic light scattering (DLS) using a Zetasizer Nano ZS (Malvern Instruments; Worcestershire, UK). Data were expressed as mean + standard deviation (SD) from three replicates.

Morphological examination of an undiluted sample of PGZ-NE was performed by transmission electron microscopy (TEM) using a JEOL JEM-1010 electron microscope (JEOL Ltd.; Tokyo, Japan). The sample preparation consisted of negative staining with uranyl acetate and 24 h of drying.

Viscosity and rheological behavior were evaluated 24 h after PGZ-NE preparation using a HAAKE RheoStress 1 rheometer (Thermo Fisher Scientific; Karlsruhe, Germany) by a program of three-step shear profile: a ramp-up period (0–50 s^−1^) for 3 min followed by a constant shear rate period at 50 s^−1^ for 1 min and a ramp-down period (50–0 s^−1^) for 3 min. The viscosity value was determined from the viscosity curve. Obtained data from the flow curve were fitted to several mathematical models in order to determine which best statistically represents the experimental data based on the correlation coefficient value (r) and chi-squared value.

The extensibility of PGZ-NE was examined in triplicate following the previously described method [22]. A sample of 0.15 g was placed on the center of the base plate of an extensometer followed by immediate placement of a glass plate (26.86 g) on top of the sample. The extended area was measured after an equilibrium period of 1 min. Afterwards, the sample was compressed by adding known weights (36.86, 46.85, 76.85, 126.83, and 226.80 g) at 30 s intervals, after which the area of the sample was recorded.

### 2.4. In vitro Release Study

The release study of PGZ from the formulation was carried out using Franz diffusion cells of 13 mL (FDC 400; Crown Grass; Somerville, NJ) filled with receptor medium that consisted of a mixture of DMSO (dimethyl sulfoxide):water (60:40, *v/v*) which was maintained at 32 °C and under continuous stirring in order to achieve the sink conditions. A dialysis membrane (MWCO 12 KDa) previously hydrated and degreased for 24 h with methanol:water (50:50, *v/v*) was washed with water and mounted between the donor and receptor compartment. The effective diffusion area was 2.54 cm^2^. Then, 0.2 mL of formulation was placed in the donor compartment. Aliquots of 300 µL were extracted from the receptor compartment and replaced with the same volume of receptor medium at predetermined time intervals over a 46 h period. The collected samples were analyzed by high-performance liquid chromatography (HPLC) in order to quantify the amount of PGZ released [23]. Results are shown as mean ± SD from three replicates. Data from the release curve were fitted to following kinetic models: first order, Higuchi, Hyperbolic, Weibull, and Korsmeyer–Peppa. The model with the best fit of the experimental data was selected based on the coefficient of determination (r^2^). Additional amodelistic parameters, including dissolution efficiency (DE) and mean dissolution time (MDT), were calculated from the experimental release curve.

DE was suggested by Khan and Rhodes (1972) as a parameter for the evaluation of in vitro dissolution [24]. This is estimated from the cumulative curve of dissolution rate using Equation (1):

(1) $$\mathrm{DE}\;\left(\%\right)=\frac{\int_{0}^{t}A_{d}dt}{A_{d100\cdot }t}\cdot 100$$

where $\int_{0}^{t}A_{d}dt$ is the area under the dissolution curve up to a time *(t)* and $A_{d100\cdot }t$ is the area of the rectangle calculated from the maximum experimental amount of drug released and the last time interval of the experiment.

MDT is the mean residence time of drug in the formulation. It is calculated using Equation (2).

(2) $$MDT=\frac{\sum \left(t_{i}\Delta A_{dt}\right)}{A_{d\infty }}$$

$t_{i}$ is the midpoint of a time interval, $A_{dt}$ is the increase in the amount of dissolved drug at the corresponding interval, and $A_{d\infty }$ is the maximum amount of dissolved drug in the experiment.

### 2.5. Ex vivo Permeation Study

Human skin of a healthy 38-year old woman obtained during an abdominal lipectomy (Hospital of Barcelona, SCIAS, Barcelona, Spain) with previous written informed consent in accordance with the Ethical Committee of the Hospital of Barcelona (number 001, dated 20 January 2016) was used to carried out the assay. The integrity of the skin samples was evaluated by Transepidermal water loss (TEWL) using a Tewameter TM 300 (Courage & Khazaka Electronics GmbH; Cologne, Germany) and those with results below 10 g/m^2^h were used. Skin samples (0.4 mm thick) were mounted between the donor and receptor compartment of Franz diffusion cell (6 mL) with diffusion area of 0.64 cm^2^. The receptor medium consisted of a mixture of Transcutol-P:water (60:40, *v/v*) which was kept at 32 °C and under stirring at 600 rpm to guarantee sink conditions. The rest of the assay was performed as described in Section 2.5.

After permeation studies, the amount of PGZ retained in the skin (Q_ret_, mg/g skin/cm^2^) was extracted by ultrasound-assisted extraction. The skin samples were removed from Franz diffusion cells and washed with distilled water. The edges were cut in order to retain only the permeation area, which was weighed and immersed in 2 mL of methanol for 20 min using an ultrasonic bath. Afterwards, the solution was filtered and analyzed by HPLC.

### 2.6. Efficacy Studies: Oxazolone-Induced Atopic Dermatitis

#### 2.6.1. Animals and Study Protocol

The efficacy studies were carried out in female hairless mice SKH-1 (Charles River Laboratories; France) that were 7–9 weeks old following a previously approved protocol by the Animal Experimentation Ethics Committee of the University of Barcelona (Generalitat de Catalunya Ref. 8756, data 28/01/2016). The mice were sensitized by topical application of 20 µL of 2.5% oxazolone dissolved in acetone on dorsal skin. Seven days later, 60 µL of 0.1% oxazolone were applied daily over a period of 16 days in order for the mice to retain the disease throughout the duration of the experiment. The sensitized mice were classified into two groups (*n* = 3): one group was treated with 30 µL of PGZ-NE (PGZ-NE group), whereas the other was treated with 30 µL of water (positive control group) for 9 days starting on Day 15 until the finalization of the experiment on Day 23. Treatment with PGZ-NE or water was applied 1 h after 0.1% oxazolone application. A negative group that was comprised of healthy mice was also used to compare the results. The mice were euthanized 8 h following the final treatment and the area of skin used in the experiment was surgically excised. Thickness of these extracted skin was measured using a Pocket Thickness Gage 7309 (Mitutoyo Corp.; Kawasaki, Japan).

#### 2.6.2. Biomechanical Skin Properties Evaluation

TEWL and SCH (Stratum corneum hydration) of the mice’s dorsal skin were evaluated at 0, 7, 15, 17, 19, 21 and 23 days prior to application of oxazolone and treatment. These parameters were measured using a Tewameter TM 300 (Courage & Khazaka Electronics GmbH; Cologne, Germany) and a Corneometer 825 (Courage & Khazaka Electronics GmbH; Cologne, Germany), respectively.

#### 2.6.3. Skin Evaluation by Atomic Force Microscopy (AFM)

Skin samples of each group were studied using an AFM Multimode IV controlled by Nanoscope V electronics (Bruker AXS Corporation; Santa Barbara, CA). Silicon AFM tips with a nominal spring constant of 42 nN nm^−1^ were used. For force spectroscopy measurements, the spring constant of each cantilever was determined using the thermal noise method.

Hairless mouse skin was defrosted at room temperature and immediately attached onto a steel disc with an epoxy resin. The surface was rinsed gently with buffer and water, and then subsequently dried with nitrogen. Each sample was directly mounted on top of the AFM scanner and imaged. Images were acquired in both air and contact mode at 0° scan angle with a scan rate of 3 Hz. The environment was maintained at 24 °C and 60% humidity. All images were processed using NanoScope Analysis Software (Bruker AXS Corporation; Madison, WI).

Young’s modulus evaluation of the skin samples was determined as a first approximation by using the Hertz model. Mechanical properties were measured by recording arrays of 32 × 32 force curves of selected regions using a maximum force of 0.5–1 nN in order to avoid sample damage with approach and retraction speeds of 1.0 µm/s and a contact time of 100 ms.

#### 2.6.4. Histological Analysis

For histological observation of skin architecture, hematoxylin and eosin staining was performed. The skin samples of each group were rinsed with Phosphate-buffered saline (PBS) and immediately fixed in 4% buffered formaldehyde at room temperature. After fixation, all samples were paraffin embedded onto cassettes, sectioned into 5 µm slices, mounted on microscope slides and stained with hematoxylin and eosin. Finally, the samples were viewed using Olympus BX41 microscope equipped with Olympus XC50 camera.

#### 2.6.5. Pro-Inflammatory Cytokines Determination

The RNA was isolated from mouse skin using RNA Pro solution (MP Bio; Solon, OH). The samples were transferred to microtubes containing ceramic beads and placed in a FastPrep-24 instrument for three cycles of 30 s, 5500 rpm. The homogenate was utilized for RNA extraction using the illustra RNAspin Mini RNA Isolation Kit (GE Healthcare; Chicago, IL) in accordance with the manufacturer’s instructions. The concentration and purity of RNA samples were evaluated by calculating the ratio of absorbance at 260 and 280 nm using the NanoDrop^®^ spectrophotometer TM 2000 (Thermo Fisher Scientific; Waltham, MA). RNA (1 µg) was reverse transcribed using the High Capacity cDNA Reverse Transcription kit (Applied Biosystems; Foster City, CA) in a final volume of 20 µL following the manufacturer’s recommendations. Quantitative reverse transcription polymerase chain reaction (RT-qPCR) was performed in a StepOnePlus PCR cycler (Applied Biosystems; Foster City, CA) by using SYBR^®^ Green PCR Master Mix (Applied Biosystems; Foster City, CA) and specific oligonucleotides for IL-6, IL-17, and TNF-α. The housekeeping β-actin gene was used as a normalizing gene (Table 1). The standard polymerase chain reaction (PCR) program was: 5 min at 94 °C for denaturalization, 30 cycles of amplification at 72 °C for 2 min, 1 min at 94 °C, 1 min at 60 °C, and a final cycle at 72 °C for 10 min for final extension. Relative gene expression was represented as fold-change compared with control and was calculated by ΔΔ Ct formula.

#### 2.6.6. Statistical Analyses

Statistical analysis was performed using SPSS (version 20.0; Chicago, IL) software package. All assays were repeated at least three times independently in triplicate. The values are presented as the mean ± SD. Differences among more than two groups were assessed using one-way ANOVA followed by Tukey’s test. The p-values less than 0.05 were considered statistically significant.

## 3. Results

### 3.1. Pseudo-Ternary Phase Diagram and Formulation

**Figure 1:**
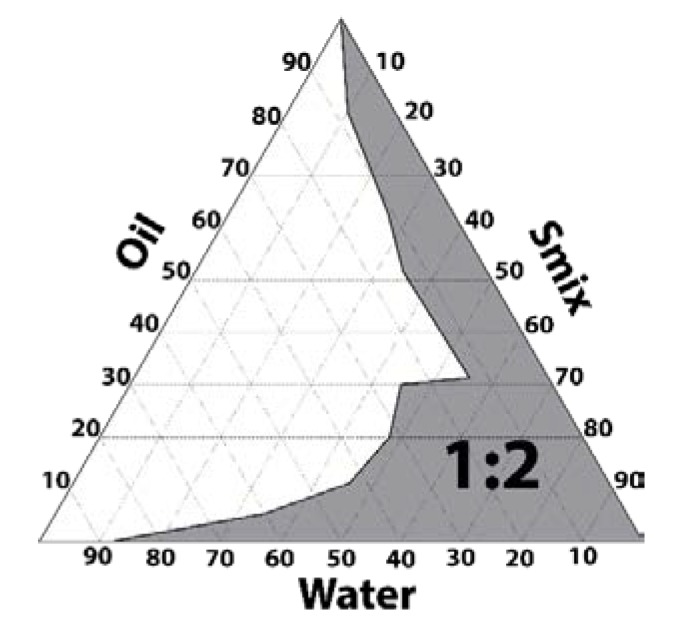
Pseudo-ternary phase diagrams: Capryol 90 (oil phase), Labrasol (surfactant), Transcutol-P (cosurfactant), and water (aqueous phase). Labrasol and Transcutol-P were mixed at the ratio 1:2 (w/w).

Pseudo-ternary phase diagram was constructed using the excipients that exhibit the greatest solubilizing potential for PGZ. Capryol 90 (oil phase), Labrasol (surfactant), and Transcutol-P (cosurfactant) show a solubilizing capacity of 1.02, 1.5, and 1.89 mg/g, respectively [21]. The obtained diagram using a mixture of Labrasol and Transcutol-P at the ratio 1:2 exhibited a great area of emulsification for the incorporation of the drug (Figure 1).

Final formulation of PGZ-NE (Table 2) was obtained by incorporating PGZ in Capryol 90 (8%), Labrasol (19%), Transcutol-P (38%), water (17%), and Pluronic F127 (18%). The addition of the triblock copolymer Pluronic F127 into NE was an uncomplicated process thanks to its amphiphilic nature, which allowed it to form a homogeneous mixture with oil, surfactant and cosurfactant of the NE.

### 3.2. Characterization of PGZ-NE

PGZ-NE had a pH value of 5.23 as well as a transparent, homogeneous, and fluid appearance with no thermoreversible gelation properties.

**Figure 2:**
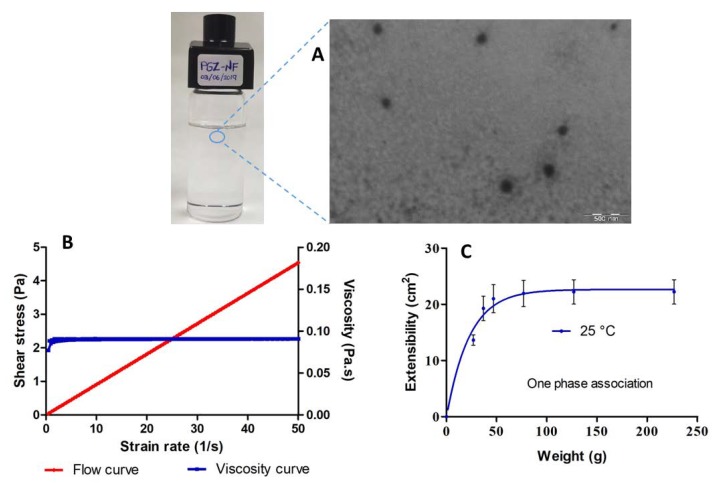
Characterization of pioglitazone-nanoemulsion (PGZ-NE): (**A**) transmission electron microscopy (TEM) image of PGZ-NE. Magnification 40,000 ×; (**B**) rheogram of PGZ-NE at 25 °C showing both flow and viscosity curves; and (**C**) extensibility profile of PGZ-NE at 25 °C.

The mean droplet size was 158.30 ± 4.67 nm with a PI value of 0.28 ± 0.06. These results were consistent with the images observed by TEM (Figure 2A), which showed the presence of nano-droplets of spherical shape and uniform distribution.

Figure 2B shows the rheological profile of PGZ-NE obtained at 25 °C. The flow curve exhibits a linear relationship between shear stress and shear rate, whereas the viscosity curve shows a constant value of 90.75 ± 0.003 mPa·s as the shear rate increased, which is characteristic of Newtonian fluids. The mathematical equation that best described experimental data (r^2^=1) was the Newton model, which consequently confirmed the Newtonian behavior of the system.

Figure 2C shows the extensibility profile of the formulation, whose values increased proportionally with loading weight until reaching a maximum extensibility of 22.2 cm^2^. According to the mathematical modeling, this formulation showed one phase association profile at 25 °C. 

### 3.3. In Vitro Release Study

**Figure 3:**
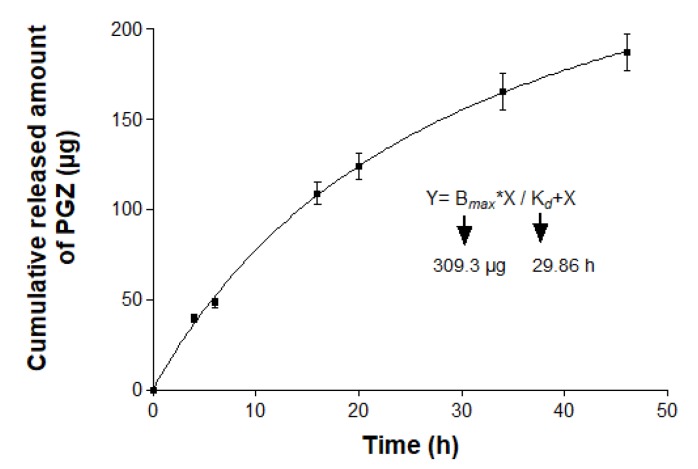
In vitro release profile of pioglitazone (PGZ) from nanoemulsion (NE). Data represented by mean ± standard deviation (SD) (*n* = 3).

The graphical representation of the cumulative amount of drug released from the formulation versus time is shown in Figure 3. After 46 h of assay, an amount of 187 µg of PGZ was released from the NE, representing 93.5% of the drug placed in the donor compartment. The best fit of the experimental data was obtained with the hyperbolic model with a r^2^ = 0.9997 and whose mathematical equation is Y=B_max_X/K_d_+X, where the amount of drug released (Y) at a certain time (X) is equal to the quotient between the maximum amount of drug released (B_max_) multiplied by X and the release constant (K_d_) multiplied by X. In this work, B_max_ was found to be 309.3 µg and K_d_ was 29.86 h. Additionally, amodelistic parameters evaluation showed values of 64.59% for the DE and 16.29 h for the MDT. These amodelistic pharmacokinetic parameters were calculated from the experimental data independent of the mathematical modeling.

### 3.4. Ex Vivo Permeation Study

The evaluation by HPLC of the aliquots extracted from the receptor compartment showed that PGZ did not permeate through human skin and thus the permeation and prediction parameters could not be calculated. However, PGZ was found inside the skin showing a high retention value of 478.08 µg/g skin/cm^2^.

### 3.5. Efficacy Studies: Oxazolone-Induced Atopic Dermatitis

Topical application of oxazolone on the hairless mice skin induced hallmarks of AD such as erythema, scaling, and edema, all of which were macroscopically evident on Day 15 of the experiment. The positive control group showed a worsening of these symptoms as oxazolone application on skin proceeded. In contrast, PGZ-NE progressively improved skin appearance from the second day of treatment. The skin lesions of this group visibly disappeared, showing healthy skin that was comparable to the negative control at the end of the experiment.

#### 3.5.1. Biomechanical Skin Properties Evaluation

**Figure 4:**
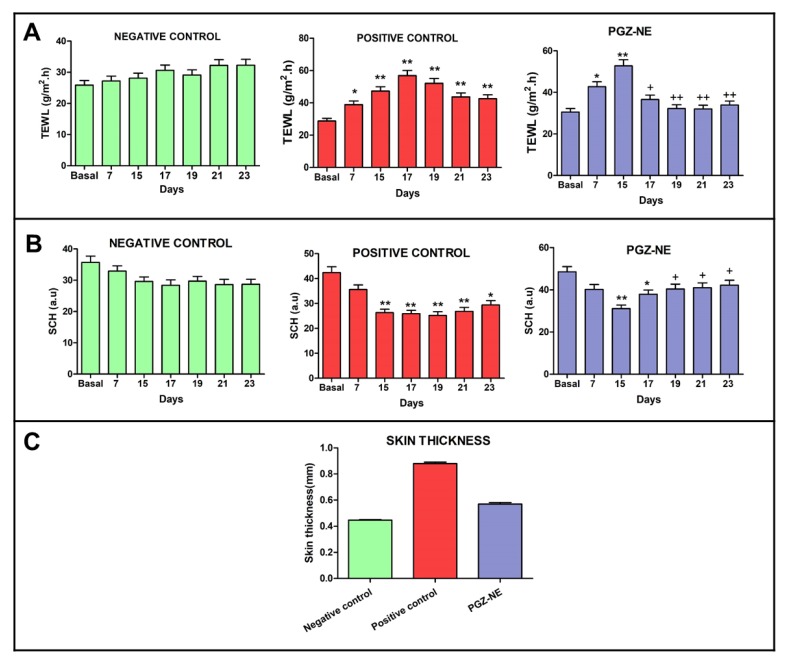
Evaluation of skin barrier function in each experimental group using oxazolone-induced atopic dermatitis model: (**A**) Transepidermal water loss (TEWL); (**B**) Stratum corneum hydration (SCH); and (**C**) skin thickness. Results are expressed as mean ± SD (*n* = 3). Significant statistical differences: * *p* < 0.05, ** *p* < 0.01 comparison with the basal state. + *p* < 0.05, ++ *p* < 0.01 comparison with Day 15 (first administration of PGZ-NE).

Topical application of oxazolone on the skin significantly increased TEWL and decreased SCH due to damage of the skin barrier, which is consistent with the lesions observed macroscopically. Despite this, the mice that received PGZ-NE by topical administration significantly reduced TEWL from the second day of treatment and increased SCH from the fourth day of treatment to the point of not displaying significant differences with respect to the basal state in both parameters (Figure 4A,B). The thickness evaluation of the skin extracted after the experiment also corroborated these findings, as a markedly greater thickness was observed in the positive control compared to the PGZ-NE group (Figure 4C). These results indicate that PGZ-NE reduces inflammation and alleviates oxazolone-induced skin lesions.

#### 3.5.2. Skin Evaluation by AFM

**Figure 5:**
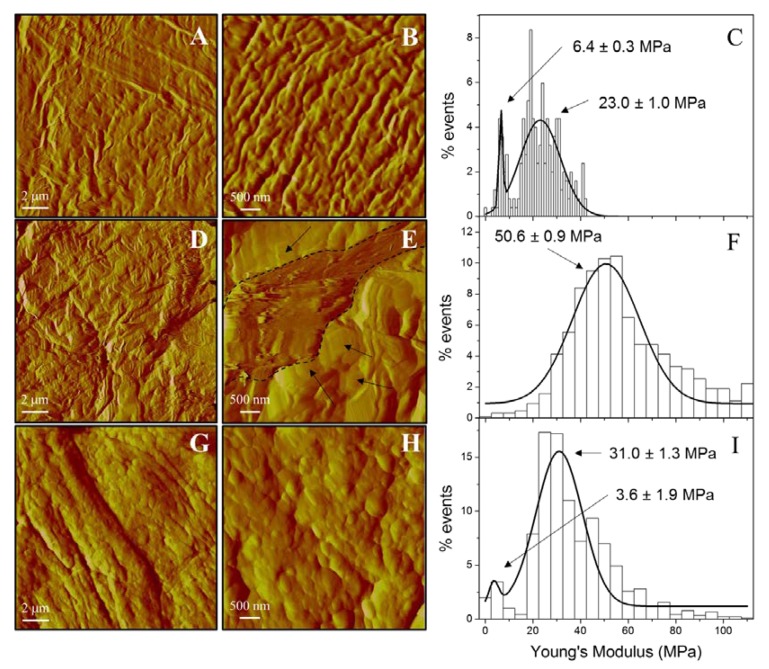
Atomic Force Microscopy (AFM) deflection error images and Young’s modulus distribution for: control skin (**A**–**C**); skin treated with oxazolone (**D**–**F**); and skin treated with PGZ-NE (**G**–**I**). The continuous lines in Young’s modulus distribution represent the fitting of the experimental data to normal distributions.

To perform an in-depth investigation of the impact of oxazolone on hairless mice skin and the reversion of the effects due to the application of PGZ-NE, the nanostructure of both healthy and damaged skin was analyzed with atomic force microscope (AFM). Figure 5 shows AFM ‘Deflection Error’ images of untreated skin (negative control; Figure 5A,B), positive control (Figure 5D,E), and skin treated with PGZ-NE (Figure 5G,H). Negative control (healthy skin) showed a wavy surface with a mean roughness value of 33 ± 6 nm while the treatment with oxazolone (positive control) prompted the presence of amorphous structures (represented by black arrows in Figure 5E) that were 0.5–1 µm in size, with hundreds of nanometers protruding from structureless regions (represented by the encircled dashed line structure in Figure 5E). After the treatment with the PGZ-NE, the skin once again displayed a wavy surface but with additional round-shaped structures on it. These round structures were 200–300 nm in size and 10–20 nm in height and, in this case, the skin was smoother with a mean roughness value of 11.0 ± 1.8 nm.

The elasticity of the skin was evaluated via Young’s modulus determination. Figure 5 depicts the distribution of Young’s modulus for the negative control (Figure 5C), positive control (Figure 5F), and post-application of PGZ-NE (Figure 5I). The mean of Young’s modulus for the negative control is 23 MPa, which is consistent with other reports on mouse skin determinations [25]. However, a second peak is observed at lower values, which may be attributed to lower adhesion of corneocytes that were eliminated as the skin surface was being rinsed. The treatment with oxazolone (positive control) provoked a rigidification of the skin because the mean of Young’s modulus is 50.6 MPa, which was more than twice the value of the negative control. Interestingly, the application of PGZ-NE reversed the rigidification because of the oxazolone, as evidenced by the reduction of the Young’s modulus value to 31 MPa.

#### 3.5.3. Histological Analysis

**Figure 6:**
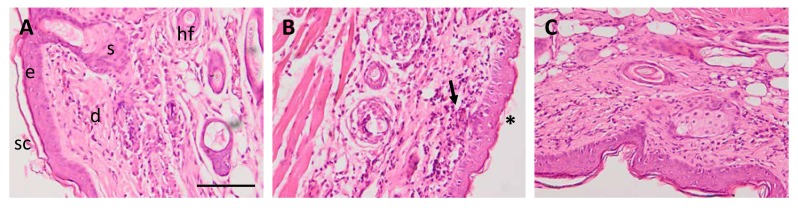
Representations of histological sections (original magnification, × 200) of mouse skin: (**A**) control conditions/negative control; (**B**) oxazolone-induced atopic dermatitis/positive control; and (**C**) PGZ-NE treatment in mice with oxazolone-induced atopic dermatitis. Skin structures: epidermis (e) dermis (d), sebaceous gland (s), hair follicle (hf), stratum corneum (sc), loss of stratum corneum (asterisk), and inflammatory cell infiltrate (black arrow). Scale bar = 100 µm.

As shown in Figure 6A, healthy skin (negative control) consisted of an epidermis with a contiguous stratum corneum and a normal appearance followed by unaltered dermis with dermal appendages, including sebaceous glands and hair follicles. Positive control (Figure 6B) showed signs of dermatitis including inflammatory cell infiltrate and loss of stratum corneum accompanied by an initial loss of dermal appendages. PGZ-NE topically applied on skin (Figure 6C) prevented the loss of stratum corneum, decreased the infiltration of inflammatory cells, and reduced the alteration of the dermal appendages.

#### 3.5.4. Pro-Inflammatory Cytokines Determination

**Figure 7:**
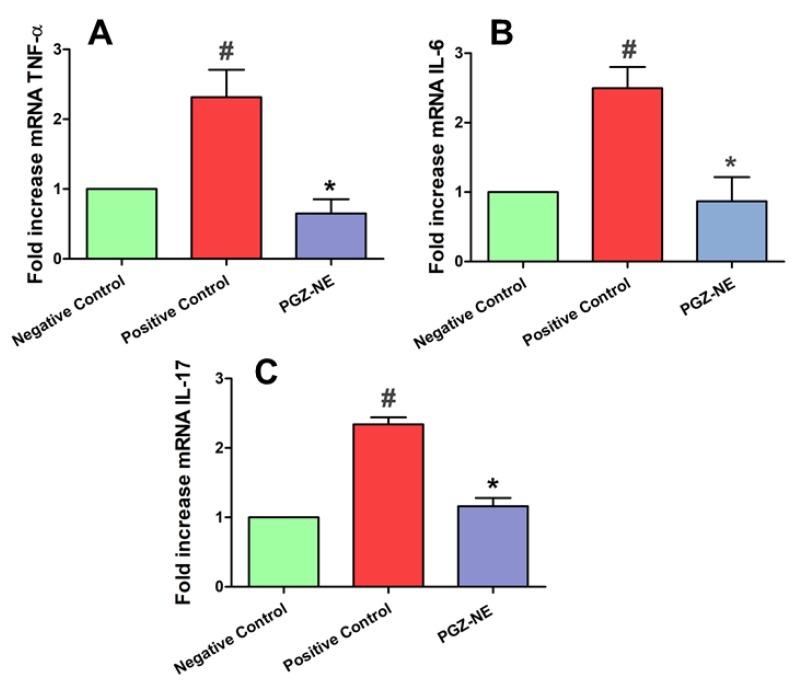
Relative expression of pro-inflammatory cytokines measured by quantitative reverse transcription polymerase chain reaction (RT-qPCR): (**A**) Tumor necrosis factor-alpha (TNF-α); (**B**) Interleukin-6 (IL-6); and (**C**) Interleukin-17 (IL-17). Negative control (healthy mice), positive control (mice with oxazolone-induced atopic dermatitis) and PGZ-NE group (mice with oxazolone-induced atopic dermatitis treated with PGZ-NE). # *p* < 0.05 negative control vs. positive control. * *p* < 0.05 positive control vs. PGZ-NE treatment.

The RT-qPCR analysis revealed a significant increase in the expression of the pro-inflammatory cytokines TNF-α, IL-6, and IL-17 in the positive control when compared to the negative group. The treatment with PGZ-NE in mice with induced dermatitis significantly reduced the expression of these cytokines to levels similar to those of the negative control. These results confirm the role of PGZ in the regulation of inflammatory processes (Figure 7).

## 4. Discussion

In this study, the incorporation of PGZ in a NE was used as a strategy to treat AD via topical application on skin. The final formulation was prepared using pharmaceutical excipients with high solubilizing potency for the drug. The oil phase was composed of Capryol 90, which is a lipophilic solubilizer consisting of a mixture of propylene glycol mono- and diesters of caprylic acid. Labrasol was used as nonionic oil/water (O/W) surfactant because it has a hydrophilic lipophilic balance (HLB) of 14. Transcutol-P was used as cosurfactant, which is an effective hydrophilic solvent that increases the solubility of both lipophilic and hydrophilic drugs [26]. Considering the high safety and biocompatibility of Transcutol-P, a ratio of 1:2 was chosen for the mixture of Labrasol and Transcutol-P in the construction of the pseudo-ternary diagram (Figure 1). Moreover, the non-ionic triblock copolymer Pluronic F127 was incorporated in the aqueous phase to increase the viscosity of the final formulation and consequently facilitate its application while increasing its residence time in the skin. This polymer exhibits amphiphilic nature, non-toxic properties, and can interact with biological membranes [27]. The final formulation of PGZ-NE (1 mg/mL) had a slightly acidic pH value, which is biocompatible with the natural acidity of the skin and thus assures non-irritating effects. The physical characterization by DLS and TEM confirmed a structure consisting of droplets of nanometric size and spherical shape (Figure 2A). This feature provides optical transparency, a large surface area, and uniform distribution on the skin. A topical formulation should have optimal organoleptic characteristics with appropriate consistency and spreadability in order to provide an aesthetically appealing vehicle for the patient that is pleasant and easy to use as well as being able to remain in the treated area for the required time. Rheology determines sensorial properties, filling/dosing behavior, spreadability, and can modulate biopharmaceutical parameters, including release rates [28]. The rheological behavior of the studied formulation was determined from the flow curve, where the relationship between shear stress and shear rate was linear whereas the viscosity remained constant. In this sense, the PGZ-NE was defined as a Newtonian fluid based on its best fit to the Newton model (Figure 2B). The study of the extensibility area provides a measure of the deformation threshold of the vehicle. A topical formulation should exhibit optimal spreadability, since a product with very high extensibility (too fluid) or with very low extensibility (too viscous) could be unpleasant or uncomfortable for patients [28,29]. The extensibility areas obtained from PGZ-NE evidence a formulation that is easy to apply and pleasant to the touch (Figure 2C).

Interactions between vehicle and drug determine the release profile, which in turn affects the subsequent percutaneous permeation. After 46 h of assay, 93.5% of the drug was released from NE (Figure 3). This result suggests that the vehicle is capable of releasing the drug and thus the formulation does not limit permeation of the drug through the skin [28]. The release kinetic of PGZ from NE followed a hyperbolic model, where a rapid release of 50% of PGZ occurred within an initial phase of 16 h and the remaining drug was slowly released during a longer second period of 30 h. In an equal manner, the amodelistic parameters evaluation that were carried out as part of the quality control confirmed an acceptable value of DE (64.59%) and MDT (16.29 h). These values are consistent with the graphic representation of the release study, in which it can be observed that the drug release curve is placed slightly above half of the rectangle represented by last time interval of the experiment (46 h) and the maximum experimental amount of drug released (187 µg).

Ex vivo permeation studies showed that the drug is retained in the tissue without reaching the receptor compartment, thereby demonstrating that PGZ-NE could be successfully used to achieve a local effect in the skin without adverse systemic effects. The high amount of PGZ retained inside the skin (478.08 µg/g skin/cm^2^) indicates that the drug can cross the SC, which is the limiting step in the permeation process from topical formulations. Consequently, it is possible to reach an effective drug concentration in the target area. This can be attributed to the properties of Transcutol-P (polar solvent) and Capryol 90 (non-polar solvents), whose combination in the formulation could result in synergetic skin penetration enhancement due to the fact that Transcutol-P increases drug solubility in the stratum corneum (SC), whereas Capryol 90 favors the diffusion of drug in the SC [30]. Additionally, Pluronic F127 enhances the diffusion ability of drug thanks to its amphiphilic structure which allows it to act as a surfactant and interact with biological membranes [31,32].

Regarding the therapeutic efficacy, the potential of PGZ-NE was evaluated using a model of oxazolone-induced AD in hairless mice that effectively reproduces the disease in clinical, histological, and immunological aspects. Oxazolone is a hapten that covalently alters skin proteins and triggers allergic responses [33]. Repeated application of oxazolone on mouse skin causes a chronic hypersensitivity reaction with multiple features of human AD including erythema, edema, erosion, and dryness [34]. In this study, these skin lesions were detected macroscopically after nine oxazolone-challenges and became increasingly pronounced in the positive control as oxazolone application advanced. Oxazolone was also applied daily in the group that received PGZ-NE in order to maintain the induced AD during the nine days of treatment with the formulation. This group notably reduced AD-like skin lesions from the second day of treatment, thereby confirming the therapeutic benefits of PGZ-NE.

In healthy skin, the external SC layer comprises a lipid-rich intercellular matrix that contains balanced amounts of ceramides, cholesterol, and free fatty acids in order to reduce TEWL and maintain the skin hydrated [35]. TEWL represents the quantity of water that diffuses through a fixed area of SC to the skin surface per unit time, and SCH is an indicator of skin dryness because it measures the water content of SC [36]. AD is characterized by having a defective or weakened epidermal barrier and lipid abnormalities that promote an increase in TEWL and a decline in SCH. Therefore, the noninvasive assessment of these biophysical parameters is successfully utilized to analyze the skin barrier function in patients with AD [37,38]. In this study, repeated application of oxazolone significantly increased TEWL and reduced SCH, indicating disruption of the epidermal permeability barrier. In the PGZ-NE group, TEWL levels increased 73.11% and SCH decreased 36.01% from the basal state after AD induction (Day 15). However, topical treatment with PGZ-NE halted the progression of the disease which then resulted in substantial improvement of these parameters with both reduced TEWL and increased SCH after two and four topical administrations of PGZ-NE, respectively. In contrast, the positive control exhibited progressive deterioration of these parameters, showing the most critical values of TEWL with an increase of 97.57% compared to the basal state while SCH decreased 40.57% (Figure 4A,B). These results are consistent with previous studies which have reported a 2–to 4-fold increase in TEWL of AD patients compared to the skin of normal controls [39]. These alterations in skin biophysical parameters during AD can be explained by the impairment of the barrier function associated with disturbed lipid composition of the SC, such as reduction in total lipids, phospholipids, ceramides, and sterol esters [40]. Skin inflammation is also characterized by infiltration of inflammatory cells as well as increased skin thickening, which is indicative of edema, increased vascular permeability and epidermal hyperplasia [41]. In this study, the positive control showed markedly greater skin thickness while its histological analysis revealed tissue lesions and presence of inflammatory markers. Conversely, topical administration of PGZ-NE aided in the improvement of these symptoms (Figure 4C and Figure 6).

AFM studies (Figure 5) have demonstrated that oxazolone modifies the skin surface structure by altering the wavy shape it displays in the negative control (healthy skin). The presence of structures that are hundreds of nanometers thick is indicative of alteration of the normal structure of the skin surface. The treatment with PGZ-NE revealed a surface resembling the negative control skin sample with some round structures found on it. Droplets are smaller than these structures, thereby suggesting that they promote a slight increase of the volume of some regions in corneocytes following its absorption into the skin. Moreover, the Young’s modulus analysis demonstrates the efficacy of PGZ-NE to revert oxazolone effects on skin, as observed in the decreased rigidity of the sample following treatment.

From the immunological point of view, AD development is characterized by the production of cytokines that greatly affect the pathophysiology of the disease. The critical role of the cytokines TNF-α, IL-6, and IL-17 in the pathophysiology of AD has been previously reported in several studies. Patients with AD show elevated TNF-α levels in the sera and skin, which is released by infiltrating mast cells, Th lymphocytes, and keratinocytes [42]. Other studies have shown TNF-α that is either alone or in combination with Th2 cytokines influences the alterations of barrier lipid properties observed in AD lesions, including the reduction of free fatty acids [43]. IL-6 is induced by immediate early inflammatory cytokines such as TNF-α and IL-1. It is expressed in the cutaneous response to allergen challenge in atopic patients and has been alternately associated with both allergic and irritant dermatitis [44]. IL-6 participates in the acute-phase response, stimulates the growth and differentiation of T and B lymphocytes, and promotes the production of IL-4, which is involved in the development of allergic immune responses [45]. IL-17 is overexpressed in chronic autoimmune disorders, AD, and asthma. Some studies suggest that IL-17 production is a common signature between AD and psoriasis. This cytokine promotes the differentiation of B cell, modulates the Th2 cellular immune response, favors the development of chronic AD form, and aggravates skin deterioration in AD by inhibiting the production of filaggrin. In our study, mice with oxazolone-induced AD showed greater than a twofold increase in the expression of TNF-α, IL-6, and IL-17 when compared with the negative control. PGZ-NE was able to reduce the production of these pro-inflammatory cytokines to levels similar to those of the negative control (Figure 7). The therapeutic benefits of PGZ-NE in the clinical, histological, and immunological levels of the AD may be due to several aspects of the NE composition, including: (i) the anti-inflammatory activity of PGZ; (ii) the protective effect of the NE droplets which adhere to the skin surface and form a film that avoids water evaporation; and (iii) the skin-repairing property of Pluronic F127, which has been reported to stimulate the proliferation of collagen fibers and the scarring in burned and scarified skin [46,47,48].

## 5. Conclusions

This study provides supporting evidence of the therapeutic benefits of PGZ-NE in the treatment of AD. This nanoformulation exhibited improved consistency by the incorporation of Pluronic F127, resulting in a fluid that is aesthetically acceptable, pleasant to the touch, physically stable, and has Newtonian behavior, which allows for easy administration by spray or roll-on. The nanoformulation was able to release the incorporated drug following a hyperbolic kinetic model as well as facilitate the passage of the drug through the stratum corneum while favoring drug retention in the skin without reaching the receptor compartment, thus guaranteeing a local effect without systemic effects as well as providing a prolonged action of the drug in clinical practice. Finally, therapeutic benefits of PGZ-NE on clinical, histological, and immunological aspects of AD were strongly demonstrated in this study with reduction of skin lesions, improvement of the skin barrier function, and decreased infiltration of inflammatory cells as well as in the expression of pro-inflammatory cytokines including TNF-α, IL-6, and IL-17. Together, these results support the notion that PGZ-NE could be used as a promising therapeutic option for the treatment of AD via topical application on affected areas.

## Figures and Tables

**Table 1:** Primer sequences used for real time polymerase chain reaction (PCR) in mouse.

Primer	Sequence (5′ to 3′)
**β-actin**	FW: GTGGGGCGCCCCAGGCACCA RV: CTCCTTAATGTCACGCACGATTTC
**IL-17**	FW: TTTTCAGCAAGGAATGTGGA RV: TTCATTGTGGAGGGCAGAC
**IL-6**	FW: TAGTCCTTCCTACCCCAATTTCC RV: TTGGTCCTTAGCCACTCCTTCC
**TNF-α**	FW: AACTAGTGGTGCCAGCCGAT RV: CTTCACAGAGCAATGACTCC

IL-17 = Interleukin-17; IL-6 = Interleukin-6; TNF-α = Tumor necrosis factor-alpha; FW = forward primer; RV = reverse primer.

**Table 2:** Final formulation of pioglitazone-nanoemulsion (PGZ-NE).

Components	(%)
Pioglitazone (1 mg/mL)	
Capryol 90 (propylene glycol monocaprylate),	8
Labrasol (caprylocaproyl polyoxyl-8 glycerides)	19
Transcutol-P (diethylene glycol monoethyl ether)	38
Water	17
Pluronic F127 (Poloxamer 407)	18
